# Ten-year follow-up after Gamma Knife radiosurgery of meningioma and review of the literature

**DOI:** 10.1007/s00701-020-04350-5

**Published:** 2020-06-26

**Authors:** Bodo E. Lippitz, Jiri Bartek, Tiit Mathiesen, Petter Förander

**Affiliations:** 1 Interdisciplinary Centre for Radiosurgery (ICERA), Radiological Alliance Hamburg, Mörkenstr.47, 22767 Hamburg, Germany; 2 Department of Clinical Neuroscience, Karolinska Institute, Centre for Molecular Medicine L8:04, Karolinska University Hospital, S-17176 Stockholm, Sweden; 3 Department of Neurosurgery, Karolinska Hospital, S-17176 Stockholm, Sweden; 4 Department of Clinical Neuroscience and Department of Medicine, Karolinska Institutet, Stockholm, Sweden; 5 Department of Clinical Medicine, University of Copenhagen, Blegdamsvej3, 2200 Copenhagen, Denmark; 6 Department of Neurosurgery, Rigshospitalet, Blegdamsvej 9, 2100 Copenhagen, Denmark

**Keywords:** Meningioma, Long-term follow-up, Gamma Knife, Stereotactic radiosurgery

## Abstract

**Objectives:**

With regard to the generally slow growth of meningioma, it is essential to analyse clinical treatment results in a long-term perspective. The purpose of the present analysis is to provide clinical data after Gamma Knife radiosurgery of meningioma in a 10-year perspective together with a review of the current literature.

**Methods:**

The current study is a retrospective analysis of 86 consecutive Swedish patients with meningiomas treated using Gamma Knife radiosurgery at the Karolinska Hospital Stockholm between March 1991 and May 2001. A total of 130 tumours were treated in 115 treatment sessions. The median radiological follow-up was 10 years (1.8–16.5 years), and the median clinical follow-up was 9.4 years (2.1–17.4 years).

**Results:**

After a median follow-up period of 10 years, local tumour control was achieved in 87.8% of meningiomas (108/123 tumours). The median latency between initial treatment and local (in-field) recurrence (*n* = 15) was 5.8 years (1.9–11.5). Recurrences adjacent but outside the initial radiation field occurred in 15.1% of patients (13/86) at a median of 7.5 years (1.3–15.7). New meningiomas were seen in 10.5% after a median of 5.4 years (0.9–10.8). In 72% of patients, no further treatment was required, 17.4% (15/86) underwent a second Gamma Knife treatment, 4.7% (4/86) required later open surgery and 5.8% (5/86) required both secondary treatments. Eighty-six percent of patients were neurologically unchanged or improved. A significantly lower rate of local (in-field) recurrences was seen in meningiomas treated with a prescription dose of > 13.4 Gy (7.1% vs. 24%, *p* = 0.02).

**Conclusions:**

The current retrospective analysis provides a 10-year follow-up and comprises one of the longest available follow-up studies of radiosurgically treated meningiomas. The current series documents a persistent high local tumour control after Gamma Knife treatment, while providing an estimation of a necessary minimum dose for long-term tumour control in meningiomas. The study confirms the validity of previous short-term data in a long-term perspective.

## Introduction

The resection of intracranial meningiomas is a classical indication for open microsurgery. The infiltrative nature of meningioma can result in structural peri-operative damage of involved vessels, sinuses and cranial nerves resulting in increased potential morbidity in cases of aggressive tumour resection. Aggressive surgical approaches have increasingly been replaced by a disease management with lower invasiveness and lower peri-operative morbidity as current priorities strictly emphasize the patient’s quality of life and avoidance of postoperative defects. ‘Noli nocere’ is the ancient term that ideally describes neurosurgery in the beginning of the twenty-first century.

The complete surgical removal of meningiomas in functional anatomical locations can be associated with a significant risk of mortality and significant postoperative neurological deficits [6, 37, 43, 55], and thus, the close involvement of eloquent or sensitive structures can make complete tumour resections virtually impossible [37]. Critical anatomical regions are the skull base, particularly the cavernous sinus and the petroclival region, but even complete resections of parafalcine meningioma can be complicated when the sagittal sinus is infiltrated. Meningiomas have often slow but highly variable growth rates with reported median doubling times ranging between 415 days and 8 years [22, 24]. Although the clinical impact of postoperative remnants is occasionally questioned, long-term studies demonstrate that incomplete meningioma resections carry a significant risk for clinically relevant tumour recurrences [24, 39].

Stereotactic radiosurgery has modified the therapeutic spectrum for meningioma and has gained an important role by reducing the risk for tumour recurrences in remnant meningiomas without significantly increasing the management risk. Stereotactic radiosurgery is more effective in smaller tumour volumes. In a successful combined management of meningioma, surgery is applied to reduce the tumour volume, while radiosurgery provides the tumour control in incompletely resected tumours. The option of a later complementary radiosurgical treatment has helped to significantly reduce the need for surgical radicality and hence the risk of potential peri-operative complications [1, 3, 42].

The Gamma Knife was the first available technology for stereotactic neuro-radiosurgery [12] with its 3D precision for the delivery of radiation still being unsurpassed. Gamma Knife radiosurgery (GKRS) of meningioma is highly effective with 5-year actuarial tumour control rates (or 5-year actuarial progression-free survival) ranging between 87 and 98.5% in 36 Gamma Knife radiosurgery (GKRS) of meningioma is highly effective with tumour control rates ranging between 70 and 98.5% in 39 Gamma Knife series comprising a total of 12,431 patients published between 2000 and December 2018 [2, 4, 5, 7, 9–11, 13, 14, 16–21, 23, 25, 26, 28, 30, 31, 34–36, 38, 44–46, 48, 49, 51, 52, 54, 56–59, 61] (Table 1). Since meningiomas are slowly growing tumours, it is paramount to validate these results in a long-term perspective. So far, 12 studies followed a total of 2523 patients for more than 71 months after radiosurgical treatment [4, 5, 9, 20, 25, 26, 31, 34, 56, 58, 59, 61], but only 5 series comprising 1364 patients covered a mean or median follow-up of more than 94 months [4, 26, 31, 34, 61].

**Table 1:** Reported tumour control after Gamma Knife treatment of meningioma

Reference	Tumour control rate at 5 years	Median follow-up (months)	Median tumour volume	Median prescription dose	Number of patients	Location
Roche et al. [48] *J Neurosurg*, 2000	92.8% (5-year actuarial progress.-free surv.)	30.5	5.8 cc (mean) (0.9–18.6 cc)	28 Gy (mean) (12–50)	92	Cavernous sinus
Aichholzer et al. [2] *Acta Neurochir (Wien)*, 2000	96% (overall tumour control rate after mean FU of 48 months)	48 (mean)	Not given	15.9 Gy (mean) (9–25 Gy)	46	Skull base
Nicolato et al. [44] *Int. J. Radiation Oncology Biol. Phys*, 2002	96% (5-year actuarial progress.-free surv.)	48.2	8.1 cc (mean) (1–20)	14.8 Gy (mean) (11–22.5 Gy)	138	Cavernous sinus
Iwai Y et al. [21] *Neurosurgery*, 2003	92% (5-year actual tumour growth control rate)		14.7 cc (mean) (1.2–101.5)	11 Gy (mean) (8–15 Gy)	43	Cavernous sinus
Flickinger et al. [10] *Int J Radiat Oncol Biol Phys*, 2003	93.2% (5-year actuarial tumour control rate)	29	5.0 cc (0.47–56.5)	14 Gy (8.9–20)	219	Various
Maruyama et al. [38] *Neurosurgery*, 2004	94.1% (5-year actuarial tumour control rate)	47	5.4 cc (0.9–39.3)	16 Gy (12–18)	40	Cavernous sinus
Kreil et al. [31] *J Neurol Neurosurg Psychiatry*, 2005	98.5% (5-year actuarial progress.-free surv.)	94.8	6.5 cc (0.38–89.8)	12 Gy (7–25)	200	Skull base
Zachenhofer et al. [61] *Neurosurgery*, 2006	94% (control of tumour growth)	103 (mean)	.	16.83 Gy (9–25 Gy)	36	Cranial base
Lee et al. [35] *Prog Neurol Surg*, 2007	93% (5-year actuarial tumour control)	.	.	13.9 Gy (mean)	964	Various (majority skull base)
Kollová et al. [28] *J Neurosurg*, 2007	97.9% (5-year actuarial tumour control rate)	60	4.4 cc (0.11–44.9 cc)	12.55 Gy (6.5–24 Gy)	325	Various
Hasegawa et al. [16] *J Neurosurg*, 2007	94% (5-year actuarial focal tumour control rate)	62	14 cc (mean)	13 Gy (mean)	115	Cavernous sinus
Iwai et al. [20] *J Neurosurg*, 2008	93% (5-year actuarial progress.-free surv.)	86.1 (mean)	8.1 cc (1.7–55.3 cc)	12 Gy (8–12 Gy)	108	Skull base
Igaki et al. [19] *Neurol Med Chir (Tokyo)*, 2009	86.9% (5-year local tumour control rate)	53.2 (mean)	3.9 cc (0.3–45)	16 Gy (12–22.5)	98	Skull base
Skeie et al. [56] *Neurosurgery*, 2010	94.2% (5-year actuarial tumour growth control rate)	82 (mean)	7.39 cc (mean) (0.40–28.9)	12.4 Gy (6–20)	100	Cavernous sinus
Flannery et al. [9] *J Neurosurg*, 2010	91% (5-year overall progress.-free surv.)	72	6.1 cc (0.3–32.5)	13 Gy (9–18 Gy)	168	Petroclival
Hayashi et al. [17] *Stereotact Funct Neurosurg*, 2011	99% (overall control in 46 months)	46	6.6 cc (mean) (0.3–50.6)	12 Gy (10–14 Gy)	66	Skull base
Santacroce et al. [49] *Neurosurgery*, 2012	95.2% (5-year progress.-free surv.)	63	4.8 cc	14 Gy	3768	Various
Starke et al. [59] *J Neurosurg*, 2012	96% (5-year actuarial progress.-free surv.)	78	5 cc (mean) (0.3–54.8)	14 Gy (mean) (8–30 Gy)	255	Skull base
Leavitt et al. [34] *Int J Radiat Oncol Biol Phys*, 2013	99% gross tumour control	123 (mean)	5.9 cc (0.1–30.4)	18 Gy (12–30 Gy)	222	Cavernous sinus
Ding et al. [8] *J Neurosurg*, 2013	70% (5-year actuarial tumour control rate)	48.6	3 cc	15 Gy	65	Parasagittal and para falcine
Starke et al. [57] *J Neurooncol*, 2014	93% (5-year Kaplan-Meier actuarial progress.-free surv.)	71 (mean)	7.8 cc (mean) (0.17–36.1)	13 Gy (5–40 Gy)	254	Petroclival
Kondziolka et al. [30] *Am J Clin Oncol*, 2016	87.7% (10-year actuarial rates of freedom from tumour progression)	56	5.5 cc	15 Gy	290	Various
Sheehan et al. [54] *J Neurosurg*, 2014	95% (actuarial progress.-free surv.)	66.7	8.8 cc (mean) (0.05–54.8)	13 Gy (5–30 Gy)	763	Sellar and parasellar
Gande et al. [11] *J Neurooncol*, 2014	95% (10-year progression-free tumour control rates)	65	8.5 cc (0.6–56.1)	13 Gy (10–20 Gy)	41	Olfactory groove
Park et al. [45] *J Neurosurg*, 2014	95% (5-year progression-free survival)	40 (mean)	3.0 cc (0.3–17.1)	13 Gy (11–16 Gy)	74	Cerebello-pontine angle
Ding et al. [7] *Neurosurgery*, 2014	95% (overall tumour control rate) 94.7% (overall 5-year progress.-free surv.)	47	3.6 cc	13 Gy	177	Cerebello-pontine angle
Jang et al. [23] *Brain Tumor Res Treat*, 2015	94.7% (actuarial 5-year progress.-free surv.)	37	.	13.9 Gy (9–19 Gy)	628	Various
Sheehan et al. [52] *J Neurosurg*, 2015	92% (actuarial 5-year tumour control rate)	60.1 (mean)	6.5 cc	13.6 Gy (8–40 Gy)	675	Posterior fossa
Sheehan et al. [51] *J Neurosurg*, 2015	90% (5-year progression-free survival)	28	5.6 cc (0.3–17.5)	15 Gy (10–20 Gy)	61	Parasagittal and parafalcine
Hafez et al. [13] *Acta Neurochir (Wien)*, 2015	95% (5-year tumour progression-free survival in 40/62 pat)	36	5.7 cc	14.4 Gy	62	Cavernous sinus
Starke et al. [58] *J Neurosurg*, 2015	88.6% (5-year progression-free survival)	78 (mean)	12.4 cc (8.1–54.8)	13.5 Gy (4.8–30 Gy)	75	Skull base (large)
Harrison et al. [14] *J Neurosurg*, 2016	93% (overall tumour control)	19.5	3.54 cc (0.2–33.8)	Range 10–18 Gy	252	Various
Cohen-Inbar et al. [4] *Neurosurgery*, 2016	88.1% (tumour volume control) 100% actuarial progress.-free surv.)	102.5	4.7 cc (0.5–23)	15 Gy (7.5–36)	135	Skull base
Hoe et al. [18] J Korean *Neurosurg Soc*, 2015	98.8% 5-year local tumour control	48	2.7 cc (0.2–10.5)	13 Gy (10–18)	320	Various
Kaprealian et al. [25] *J Neurooncol*, 2016	87% (5-year freedom from progression WHOI)	75.9	3.6 cc (0.7–35)	15 Gy (10–20 Gy)	264	Various
Lee et al. [36] *Clin Neurol Neurosurg*, 2016	92.1% (overall control)	46.1 (12–120)	0.57 (0.12–1 cc)	13.3 Gy (mean) (10–20 Gy)	113	Various
Kim et al. [26] *Clin Neurol Neurosurg*, 2017	92.2% (60/771) (overall control)	118.9 (mean) (36–180)	3 cc (mean) (2.6–6.9)	12.6 Gy (10–17 Gy)	771	Various
Cohen-Inbar et al. [5] *J Neurosurg*, 2018	91.5% (overall tumour volume control)	71	5.6 cc (0.2–54.8 cc)	14 Gy (5–35 Gy)	189	Parasellar
Patibandla et al. [46] *World Neurosurg*, 2017	83.4% (overall tumour control rate)	66 (imaging FU)	4.9 cc (0.3–105 cc)	14 Gy (5–35 Gy)	219	Central skull base

The current retrospective consecutive cohort study reports the 10-year follow-up of meningioma patients treated with Gamma Knife radiosurgery according to current clinical and technical standards. The emphasis lies on the clinical and radiological long-term outcome of this radiosurgical approach that mostly had been combined with previous open tumour resections.

## Patients and methods

All records of Swedish patients with meningiomas undergoing Gamma Knife radiosurgery between March 1991 and May 2001 at the Department of Neurosurgery at the Karolinska Hospital Stockholm, Sweden, were thoroughly reviewed. These patients were included in the long-term follow-up. Patients with anaplastic or atypical meningiomas were excluded from the current study. Hence, when histology was available, all radiosurgically treated meningiomas in the current study had been classified according to WHO grade I.

### Radiosurgical treatment

The treatment was carried out using a 201 source Cobalt-60 Leksell Gamma Knife Model B (Elekta AB, Stockholm, Sweden). In all cases, a stereotactic frame was applied under local anaesthesia followed by a gadolinium-enhanced stereotactic MRI scan. The tumour outline was delineated on the T1-weighted scans, which were imported into the planning software (Leksell Gamma Plan). The tumour margins including critical anatomical structures were outlined, and the dose plan was created with isodoses, prescription doses and maximum doses being determined by the responsible neurosurgeon in accordance with a radiosurgically trained medical physicist. The treatment protocol required that the prescription dose generally comprised more than 95% of the identified tumour volume. The contrast-enhancing dura adjacent to the meningioma, the so-called dural tail, was not included in the radiosurgical treatment field within the prescription isodose.

### Follow-up

Clinical follow-up information was gathered by retrospective review of detailed patient records. In addition, patients were contacted by letter or occasionally by phone and asked to provide structured follow-up information based upon a questionnaire.

MRI data and clinical data were reviewed retrospectively as part of the clinical routine. In general, patients were followed with annual MRI in the first 5 years after radiosurgery and with bi-annual MRI thereafter, in very few exceptions with CT scans. These images as well as the radiological reports were used for the assessment of local tumour control after radiosurgery.

### Definition of types of recurrences

The ‘radiation field’ was defined as the tumour volume contained within the prescription dose. Tumour (in-field) recurrence was defined as a progression of tumour volume within the initial radiation field, i.e. within the prescription dose. Out-of-field recurrence was defined as tumour progression immediately adjacent to the radiation field and hence outside the initial prescription isodose. The occurrence of a new meningioma was defined as a distant tumour unrelated to the radiation field.

### Statistical analysis

Kaplan-Meier plots were used to estimate actuarial growth control rates. Additional comparisons applied the Fisher’s exact test as appropriate.

## Results

### Radiosurgical treatment

Between March 1991 and May 2001, a total of 86 consecutive Swedish patients with benign meningiomas were treated with Gamma Knife radiosurgery at the Department of Neurosurgery at the Karolinska Hospital Stockholm, Sweden. During the observation period, 20 patients were treated with additional Gamma Knife sessions and a total of 130 tumours were treated in 115 treatment sessions. There were 66.2% (86/130) skull base meningioma, 17.7% falcine meningioma (23/130), 14.6% (19/130) convexity meningioma and 2 (1.5%) intraventricular meningioma. The median age at initial Gamma Knife treatment was 55 years (12.3–83.6 years). There were 61 female and 25 male patients.

The median tumour volume at the time of radiosurgery was 2.5 cm^3^ (range 0.05–50.4 cm^3^). Tumour growth prior to radiosurgery was documented in 54%. The median prescription dose was 15 Gy (7–35 Gy), and the median maximum dose was 30.7 Gy (17–70 Gy).

Before radiosurgery, 76.7% of patients (66/86) had undergone an open tumour resection in various neurosurgical centres and 57.6% of the operated patients (38/66) had developed new neurological symptoms that were related to the surgical resection.

### Follow-up

The median radiological follow-up period after initial GKRS was 10 years (1.8–16.5 years) (including deceased patients). In 3 meningiomas, the follow-up period was considered too short after secondary treatment, and in 4 meningiomas, radiological follow-up was unavailable. Hence, conclusive radiological follow-up was available in 123 meningiomas (94.6%). Ten percent of the patients had a radiological or clinical follow-up of less than 5.7 years and 5.6 years, respectively. The patients were followed clinically for a median of 9.4 years (2.1–17.4 years) with clinical follow-up being available in 95.3% (82/86).

### Local tumour control, out-of-field recurrences and remote new meningiomas

**Fig. 1:**
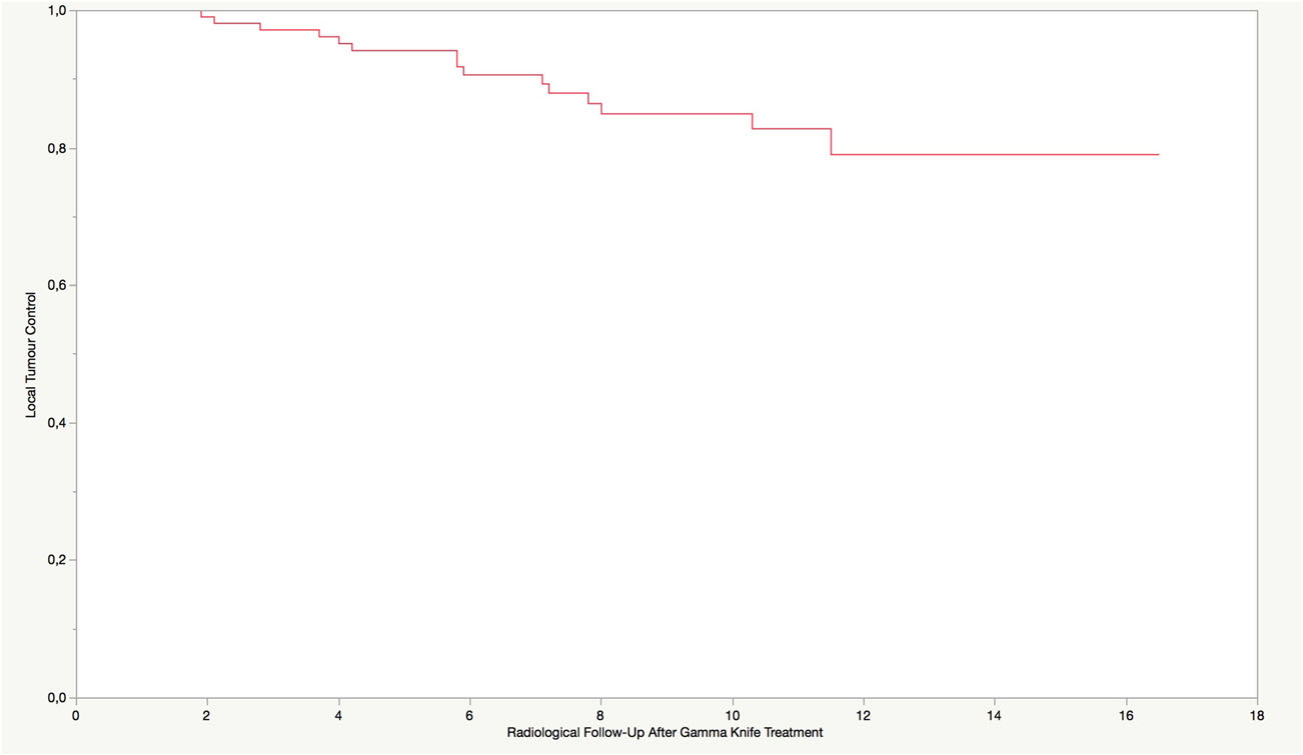
Kaplan-Meier plot: risk for local (in-field) tumour progression. X-axis: Radiological Follow-up after Gamma Knife Treatment (in years)

**Fig. 2:**
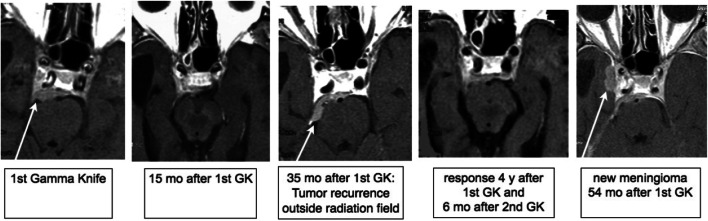
Out-of-field recurrences after Gamma Knife radiosurgery of a meningioma initially originating from the clivus. Example for a long-term response and local tumour control within the radiosurgically treated target. A recurrence outside the initial radiation field developed from the tumour’s ‘dural tail’, which is generally not included in the radiosurgical treatment field. The patient was retreated with Gamma Knife resulting in tumour regression even in the recurring/progressive parts but developed a further ‘out-of-field recurrence’ within the right cavernous sinus 54 months after the initial treatment

**Fig. 3:**
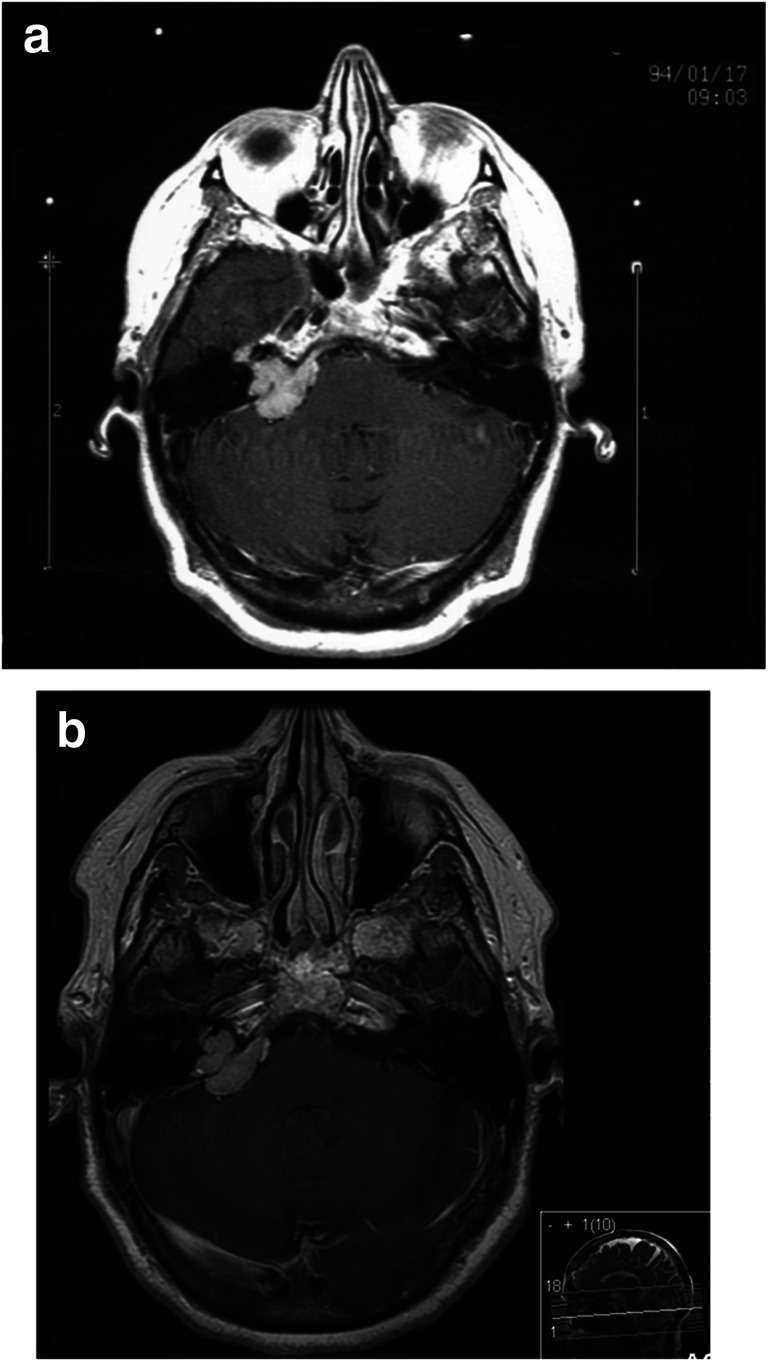
**a** Gamma Knife treatment of petrous meningioma with clival extension. **b** Follow-up 15 years after Gamma Knife treatment with virtually unchanged volume of the meningioma

After a median follow-up period of 10 years, local tumour control was achieved in 87.8% of meningiomas (108/123 tumours). There were 12.2% (15/123 tumours) (in-field) tumour recurrences in 14 patients. The median time between initial treatment and retreatment for recurrence (*n* = 15) was 5.8 years (1.9–11.5 years) (Fig. 1). In 15.1% of patients (13/86), out-of-field recurrences were documented at a median of 7.5 years (1.3–15.7) (Fig. 2). New meningiomas were seen in 12.8% of treated patients (11/86) after a median of 5.4 years (0.9–10.8). While 72% of patients (62/86) did not require any further treatment, 17.4% (15/86) underwent a second Gamma Knife treatment, 4.7% (4/86) required later open surgery and 5.8% (5/86) required both radiosurgery and open surgery (Fig. 3).

### Doses and recurrences

The risk for local (in-field) tumour recurrences was significantly increased when prescription doses lower than 13.4 Gy had been applied (24.2% vs. 7.1%; Fisher’s exact test: two-tailed *p* = 0.02). The risk for tumour recurrences was 28% for men (7/25) and 12.1% for women (7/58) (*p* = 0.1). For men whose meningiomas had been treated at prescription doses of less than 13.4 Gy, the (in-field) recurrence rate was 50% (4/8), whereas for women treated at lower doses, the recurrence risk was 15.4% (4/26) (n.s.).

### Neurological status

A total of 87.8% of patients (72/82) confirmed an unchanged or improved clinical status after a median clinical follow-up of 9.4 years (2.1–17.4 years) with complete clinical follow-up information available in 95.3% (*n* = 82). A clinical deterioration associated with the meningioma occurred eventually in 12.2% (10/82). In 7 out of 10 patients with later clinical deterioration, the symptoms were related to a tumour recurrence (5 local (in-field) recurrences and 2 out-of-field recurrences) while 3.7% of patients with available follow-up (3/82) developed side effects associated to the Gamma Knife treatment (Table 2). One of these patients presented with confusion as a result of an adverse radiation effect after a re-treatment of a recurrent clinoid process meningioma.

**Table 2:** Patients with secondary clinical deterioration after stereotactic radiosurgery

New symptoms	Time after GK	Symptom occurring at recurrence?	Specific circumstances
Pituitary insufficiency	7 years	Yes	Incomplete treatment low-dose, pre-existing compressed chiasm
Periodic disorientation	7 years	Yes	Age 88 years; ARE after retreatment
Epileptic seizure	3 years	No	Died 8.5 years after RS, (unknown reason)
Recurrence and death	6 years	Yes	Meningiomatosis 4 resections 3 GK
Focal seizures, death	8 years	Yes	Died with 91 years
Tetraparesis due to tumour progression	3 years	Yes (out-of-field)	MIB index > 20%
Unilateral loss of hearing	3 years	Yes	
Died with out-of-field recurrence	2 years	Yes (out-of-field)	Highly cellular multiple meningioma
Ophthalmoplegia	1.5 years	No	ARE (Patient died for unknown reasons)
Seizure	0.5 years	No	Multiple sclerosis

### Mortality

A total of 18 patients deceased during the long clinical follow-up. There was no short-term treatment-related mortality (e.g. > 1 year following GKRS) and no radiation-related mortality. In 8 patients, death was confirmed to be unrelated to the meningioma. In 4 patients, mortality was attributed to late meningioma recurrences:

1. 9 years after the first of five resections and 5 years after the last of three Gamma Knife treatment with signs of tumour progression of a petroclival meningioma.
2. At the age of 91, 15 years after the first operation and 8 years after radiosurgery with an extensive meningioma progression.
3. At the age of 73 with tumour progression 6 years after the first resection and 3.3 years after radiosurgery (MIB index 20%).
4. At the age of 77, a patient died as a result of a new and untreated tumour progression that had developed quickly with a volume of 19 cm^3^, which had then been considered too large to be treated with radiosurgery, 4 years after the first operation and 2 years after two Gamma Knife treatments for four and five highly cellular but non-atypical meningiomas.

In further three patients where the actual cause of death could not be established, a potential relation to the treated meningioma could not completely be ruled out:

1. One patient with unknown cause of death died 15 months after radiosurgery of a large (8 cm^3^) meningioma of the foramen magnum at the age of 78.
2. One patient had been retreated for a recurrence, had developed an ARE and died 2 years and 4 months after the second and 9 years after the first radiosurgical treatment with no information concerning the actual cause of death at the age of 89.
3. Another patient with a large meningioma that had been treated incompletely with a low dose (prescription dose 11 Gy) experienced a later recurrence and a pituitary insufficiency 7 years after Gamma Knife treatment and died due to unknown reasons.

In further 3 patients, the cause of death was unknown.

## Discussion

The present study provides the outcome data of patients with benign meningiomas treated with stereotactic Gamma Knife radiosurgery with a median radiological follow-up of 10 years (1.8–16.5 years) and a median clinical follow-up of 9.4 years (2.1–17.4 years) and provides evidence for the efficacy of radiosurgery in a long-term perspective. This is among the longest follow-up studies available in the literature of radiosurgically treated meningioma [2, 4, 5, 7, 9–11, 13, 14, 16–18, 20, 21, 23, 25, 26, 28, 30, 31, 35, 36, 38, 44, 45, 48, 49, 51, 52, 54, 56–59, 61] documenting a local tumor (in-field) control of 87.8%, which is slightly lower than control rates that have been published in other studies with a shorter observation time: in 39 Gamma Knife series comprising a total of 12,431 patients published between 2000 and 2018, the meningioma control rates ranged between 70 and 98.5% [2, 4, 5, 7–11, 13, 14, 16–21, 23, 25, 26, 28, 30, 31, 34–36, 38, 44–46, 48, 49, 51, 52, 54, 56–59, 61] (Table 1). The analysis of 3768 meningiomas in the European retrospective multicentre meningioma study documented 5-year progression-free survival rates of 95.2% [49].

Generally, local control rates are slightly lower in series with longer observation periods [27] and are very similar to the tumour control of 87.8% at 10 years found in the present study. Cohen-Inbar reported virtually identical local tumour control in 88.1% in a series with a median follow-up of 102 months [4], while the North American Gamma Knife Consortium published actuarial progression-free survival rates of 84% at 10 years after Gamma Knife treatment of petroclival meningiomas in a multicentre study of 254 patients [57].

The limitations of the current study lie in the limited total number of patients and in the fact that many of the meningiomas had been treated in the early phase of the development of radiosurgery with treatment regimens and dose planning systems that were significantly less sophisticated than currently available Gamma Knife techniques .

With an MR follow-up of 10 years, however, the current retrospective analysis comprises one of the longest available follow-up investigations in a larger series after stereotactic radiosurgery of meningiomas. It documents a persistent high local tumour control after Gamma Knife treatment, which is only slightly lower than in published observations with shorter follow-up.

### In-field recurrences and out-of-field recurrences

Meningioma recurrences within the radiation field should be differentiated from recurrences outside the initial radiation field. In-field recurrences ultimately represent intended treatment parameters, which potentially can be optimized, whereas recurrences outside the initial radiation field reflect the tumour biology and potentially progression of undetected tumour residuals. It can be argued that out-of-field recurrences can potentially be avoided through more sensitive and specific imaging and hence inclusion of tumour tissue adjacent to the outlined main treatment target. For example, imaging with Ga-DOTATOC might hold promise for sensitive targeting [33]. In contrast, remote meningiomas that appear many years after treatment must be considered as de novo tumours and unpredictable at initial radiosurgery.

While the treatment cannot prevent these late recurrences, the patient’s later management has to take this potential risk into account. The general policy behind the current study was based on the availability of MR follow-up, and the resulting clinical judgement that an eventual re-treatment for a documented tumour development outside the initial radiation field would carry a lower risk for side effects that the prophylactic inclusion of larger areas of potential and generally ill-defined ‘dural tails’.

The current study demonstrates meningioma recurrences within the treated volume in 12.2% and out-of-field-recurrences in 15.1% of patients. This relatively high risk for development of meningioma outside the initial treatment area is generally not reflected by the current literature. Some late phenomena may be underestimated in the present literature since so far only 5 out of 39 published Gamma Knife series with a total of 1364 out of 12,431 published patients reported a follow-up of more than 94 months after radiosurgical treatment [4, 26, 31, 34, 61]. The present long-term series documents new remote meningiomas in 12.8% after a median of 5.4 years and a median time span of 7.5 years between the initial radiosurgical treatment and recurrences outside the initial radiation field. This latency is reflected by another recent long-term series by Kondziolka and colleagues who reported tumour growth adjacent to the treated volume at a median of 62 months after radiosurgery [30].

The current study demonstrates a latency of 5.8 years between GKRS and in-field recurrence, which is almost identical to the 59.8-month mean latency to local recurrence as documented in a long-term series by Skeie et al. [56] in which 67% of recurrences progressed within the first 2.5 years [56]. The present results show that in-field recurrences and out-of-field-recurrences are rare and appear late, nevertheless having an important impact on clinical routines.

A long-term management is paramount for patients with meningioma where multiple interventions are often unavoidable. In the present study, 76.7% of patients had undergone an open tumour resection before radiosurgery and 17.4% underwent a second Gamma Knife treatment, 4.7% required later open surgery and 5.8% required both secondary surgery and a second Gamma Knife treatment. Since multiple interventions can be necessary, each intervention should be performed at the lowest invasive level. Long-term follow-up is absolutely essential in meningioma as the disease must be considered to be a chronic condition in many patients.

### Tumour size reduction

Volume changes after stereotactic radiosurgery of meningioma are commonly moderate. The necessity for a reduction of tumour volume is not a central issue in the radiosurgical management of meningioma, since radiosurgery should generally be avoided in large meningiomas or when symptoms result from the tumour’s mass effect. In larger meningiomas, a surgical resection remains the treatment method of choice. In cases where the tumour volume does not cause symptoms, a potential further volume reduction would be insignificant for the clinical outcome as long as further tumour progression is prevented. In these cases, the meningioma should be treated with stereotactic radiosurgery.

Size reductions were quantified in a study from the Mayo Clinic by Morita and colleagues with a typical distribution: 8% of the 88 meningiomas decreased in volume by more than 25%, 60% decreased by less than 25% and 29.5% remained unchanged [41]. Similarly, a report by Hayashi and colleagues documents a more than 50% volume reduction in 23%, and lower volume reduction in 59% and stabilization of the tumour in further 17% [17]. Shrinkage rates were significantly correlated to the amount of radiation energy delivered per tumour volume [17]. Others reported that tumours decreased in 46% and were unchanged in 44% of cases [9]. A report from Pittsburgh demonstrated a median 40% meningioma volume regression in 67% of patients and tumour stabilization in 26% [14]. Meningiomas that regressed demonstrated an 18% decrease in the first 3 months post-SRS with regression stabilizing after approximately 6 months, but a transient enlargement was observed in 9% of tumours that ultimately regressed [14]. A summary of reported tumour volume changes is provided in Table 3.

**Table 3:** Tumour volume changes after Gamma Knife radiosurgery of meningioma in published series

Tumour shrinkage (%)	Tumour stable (%)	Number of patients	Reference
46%	44%	168	Flannery et al. J *Neurosurg*, 2010 [9]
27.2%	55.4%	92	Roche et al. *J Neurosurg*, 2000 [48]
46%	47%	108	Iwai et al. *J Neurosurg*, 2008 [20]
58%	34.5%	3768	Santacroce et al. *Neurosurgery*, 2012 [49]
63%	34%	138	Nicolato et al. *Int J Radiat Oncol Biol Phys*, 2002 [44]
52%	44%	46	Aichholzer et al. *Acta Neurochir (Wien)*, 2000 [2]
69.7%	27.8%	368	Kollová et al. *J Neurosurg*, 2007 [28]
82%	17%	66	Hayashi et al. *Stereotact Funct Neurosurg*, 2011 [17]
33%	64%	36	Zachenhofer et al. *Neurosurgery*, 2006 [61]
67%	26%	252	Harrison et al. *J Neurosurg*, 2016 [14]

### Side effects

In the present series, a total of 87.8% of patients (72/82) were neurologically unchanged or improved. The data revealed a clinical long-term management risk of 12.2%, which was higher than reported in comparable series with short-term follow-up, but the majority of side effects (7/10) were unrelated to the radiosurgical treatment but appeared late and were associated with tumour recurrences (5 in-field recurrences and 2 out-of-field recurrences). Only 3.7% of patients (3/82) with available follow-up developed side effects that were directly related to the Gamma Knife treatment (Table 1). Hence, the risk for treatment-related side effects was low. Similar to the present study, Starke and colleagues reported that tumour progression was present in 64% of patients with new or worsening neurological decline [58].

After linear accelerator–based radiosurgery of benign meningioma, the 5-year actuarial rate for the development of post-radiosurgical symptoms was 26.0%, which appears to be relatively high [32]. After Gamma Knife treatments, Kondziolka reported in a long-term study that 94% asymptomatic patients remained asymptomatic [30]. Other studies reported transient radiosurgical sequelae in 3.5% and permanent side effects in 1% [44], but in general, the reported risk for clinical side effects after Gamma Knife treatment ranges between 4 and 8% [10, 28, 48, 49, 53, 57, 61].

### Adverse radiation effects

In serial structures such as the optic nerve, side effects are predominately related to dose thresholds, while a parenchymal radiation-induced tissue irritation is related to the applied dose and the volume of the irradiated brain tissue. The consequence of a radiation-induced tissue irritation is the occurrence of adverse radiation effects (AREs) that rarely occurs in radiosurgical treatment of meningiomas, as the applied radiation doses are relatively low.

AREs are seen on T1-weighted MRI images as secondarily increasing oedema and also as ring-shaped peripheral contrast enhancement. The radiation-induced oedema in meningiomas appears to occur late with the highest risk at about 11 months after SRS [18], is transient in most cases and generally regressing 18 months after SRS [51]. The clinical manifestation depends on the anatomical location of the secondary oedema. In the present study, only two patients presented with clinically symptomatic ARE, one of these patients after a second stereotactic radiosurgical treatment. Hence, the present study does not contribute data for the description of ARE in meningioma.

One early long-term study comprising patients who had been treated in the 1990s and published in 2001 showed transient radiation-induced oedema in 10.3% after Gamma Knife treatment at a frequency that must be considered as unusually high according to present standards [27], but 8/9 patients who had developed oedema had been treated at higher prescription doses [27]. Lee and colleagues who had summarized the radiosurgical experience in Pittsburgh after treatment of 964 patients with meningioma noted that the incidence of adverse radiation effect ranged from 5.7 to 16%, but that side effects were gradually reduced with better imaging and lower dosing [35]. In small-sized meningiomas, peri-lesional oedema occurred in 6.1% [36]. The large study from the Hospital Na Homolce in Prague published by Kollova and colleagues had demonstrated peri-lesional oedema after radiosurgery in 15.4% and temporary and permanent morbidity rates of 10.2% and 5.7%, respectively [28]. Virtually the same rate for the risk of oedema (15%) was recently reported in large series by Jang (15%) [23] or by Hoe (15.3%) [18].

### Tumour volumes

As larger tumours are associated with a higher risk for radiation-induced oedema, the tumour volume is generally seen as the most complicating factor in stereotactic radiosurgery. A recurring question concerns the largest possible volume that can be treated with stereotactic radiosurgery. This volume may differ depending on the tumour location. Hoe and colleagues found increased management risks above relatively low tumour volumes of 4.2 cc [18]. Petroclival meningiomas with volumes of 8 cc and larger showed a significantly increased risk for tumour progression [9], and similarly in an earlier series, the outcome after Gamma Knife treatment was significantly worse in parasagittal meningiomas larger than 7.5 cc [29]. In cavernous sinus meningioma, the complication rate was considerably higher (21% vs. 3%) in meningiomas larger than 9.4 cc [47].

### Pre-treatment oedema

Pre-treatment oedema and hemispheric tumour location have been associated with an increased risk for peri-tumoural oedema after radiosurgery [18, 50]. In some extreme cases, pre-radiosurgical oedema in convexity, parasagittal or falcine meningiomas was even associated with the occurrence of severe persistent [51] secondary oedema after Gamma Knife treatment. In a series by Hasegawa, 4 out of 6 patients with pre-radiosurgical oedema from convexity, parasagittal or falcine meningiomas developed severe panhemispheric oedema after GKRS [15]. Based on these studies, pre-existing oedema should be considered a relative radiosurgical contra-indication as the oedema can increase significantly and can persist after radiosurgery.

### Dose threshold for tumour control

The necessary dose threshold for successful tumour control in meningiomas remains to be defined, but the current series allowed an estimation of a necessary minimum dose (of 13.4 Gy) for tumour control in meningiomas while validating and confirming the short-term data from previous series in a long-term perspective. The European multicentre Gamma Knife study documented effective control in 92.5% of 3768 evaluated meningiomas that had been treated at a median prescription dose of 14 Gy [49]. The present long-term study demonstrated that patients who had been treated at prescription doses less than 13.4 Gy showed a significantly higher risk for a local recurrence of the meningioma (24.2%). At higher prescription doses, the risk for recurrence was only 7.1%. Similarly, tumour margin dose below 13 Gy significantly increased the likelihood of tumour progression in 763 patients with sellar or parasellar meningiomas treated with GKRS [54]. Kollova observed that a significantly higher incidence of tumour volume increase occurred in meningiomas treated with a margin dose lower than 12 Gy [28]. Skeie reported that in cavernous sinus meningiomas, lower prescription doses of 11.5 Gy were significantly associated with further tumour growth [56] and the team from Charlottesville noticed an increasing risk for tumour progression with decreasing dose to tumour margin [59]. On the other hand, Iwai et al. published the long-term outcome after Gamma Knife treatment of 108 patients and proposed to use lower prescription doses ranging from 8 to 12 Gy (median 12 Gy) [20]. With these lower doses, the actuarial progression-free survival rate was 93% at 5 years and 83% at 10 years, which is not different from other series applying higher doses [20].

With regard to the results from the present long-term study and the data cited above, it is safe to claim that meningiomas should be treated at prescription doses above 13–14 Gy for an improved chance to achieve long-term tumour control.

### Gender differences

In the present long-term study, the risk for tumour recurrences was 28% for men and 12.1% for women (7/58). Due to the relatively low numbers, however, the difference was not significant. It is interesting, however, that men who had been treated at low prescription doses (< 13.4 Gy) carried a 50% risk for local recurrence of the meningioma. The present numbers are too small to draw conclusions based on this study alone, but serve as supporting evidence with regard to similar results that had been demonstrated by others: Kollova and colleagues had reported a lower local tumour control in male patients with meningioma [28], and the European multicentre meningioma study found that significantly higher tumour control in female patients [49]. Similarly, multivariate predictors of favourable outcome included female gender in the multicentre study of benign petroclival meningioma from the North American Gamma Knife Consortium [57], and male sex was a significant risk factors for tumour progression [25] in petroclival meningiomas and cerebello-pontine angle meningiomas [7, 9].

### Cavernous sinus and skull base meningiomas

In a large series of 255 patients with skull base meningiomas treated with Gamma Knife, the actuarial progression-free survival at 5 and 10 years was 96% and 79%, respectively [59], while Igaki documented actuarial local tumour control rates of 86.9% and 78.9% at 5 and 10 years, respectively [19]. Higher local control was seen in smaller tumours (≤ 4 cc) and in meningiomas treated with prescription doses above 14 Gy [19]. New cranial neuropathies occurred or worsened in 8.6% and decline in cognition or memory or cerebellar deficits, etc. in 2%, with petrous or clival location being predictive factors for side effects versus parasellar, petroclival and cerebello-pontine angle location [59]. In cavernous sinus, meningiomas local tumour control rates were 99% at 5 years [47] and 90.4% at a mean follow-up of 82.0 months with a resulting 10-year actuarial tumour growth control rate of 83.8% [56]. The complication rate of 6% included optic neuropathy, worsened diplopia or pituitary dysfunction while 21.0% of patients experienced improvement of symptoms [56].

### Sellar and parasellar meningiomas

Due to their involvement of neurovascular and endocrine structures, complete resection of parasellar and sellar meningiomas can be associated with significant morbidity and incomplete resections are common. A multicentre study of ten centres of the North American Gamma Knife Consortium of patients identified 763 patients with benign sellar and parasellar meningiomas with median follow-up of 66.7 months. At the last follow-up, tumour volumes remained stable or decreased in 90.2% of patients with 88% actuarial progression-free survival rate at 8 years with new or worsening cranial nerve deficits occurring in 9.6% and additional 4.2% of patients experiencing other forms of symptom progression [54].

### Posterior fossa meningioma and cerebello-pontine angle meningiomas

The large multicentre study of 675 patients treated with Gamma Knife radiosurgery for posterior fossa meningiomas documented tumour control in 91.2% at a mean follow-up of 60.1 months with resulting actuarial 10-year tumour control of 81% and a total of 27.4% of patients showing improvement in clinical outcome [53]. Trigeminal dysfunction was the most frequent new or deteriorated cranial nerve symptom followed by dysfunction of Nn. III/IV/VI [53]. Clival tumour locations, petrous or CPA locations rather than petroclival, tentorial and foramen magnum locations were predictive of neurological deterioration [53]. A virtually identical outcome was reported from the multicentre study of cerebello-pontine angle meningiomas with actuarial rates of progression-free survival of 93% and 77% at 5 and 10 years, respectively, with permanent neurological deterioration occurring in 8.5% with most common worsening neurological deficits being dizziness, imbalance, hearing loss or permanent trigeminal nerve dysfunction in 4% (transient in 54.5%) [7].

### Petroclival meningiomas

In a large multicentre study of 254 patients with benign petroclival meningioma, 140 patients had been treated with upfront radiosurgery while 114 patients were treated with Gamma Knife following surgery. Kaplan-Meier actuarial progression-free survival rates at 10 years were 84%, and at last clinical follow-up, only 6.4% of patients had experienced progression of symptoms [57]. Similarly, Flannery, Kondziolka and colleagues had reported 10-year progression-free survival rates of 86% in their series with petroclival meningiomas with meningiomas larger than 8 cc having a significantly increased risk for tumour progression [9].

### Parasagittal and parafalcine meningiomas

Neurological function did not deteriorate, and no additional therapy was required in patients after Gamma Knife treatment of parasagittal meningiomas smaller than 7.5 cc [29]. The experience at the University of Virginia with Gamma Knife treatment of parasagittal and parafalcine meningiomas (WHO grade I) was published in two different cohorts: 65 patients with 90 meningiomas with median treatment volume of 3.7 cc (range 0.7–33.1 cc) treated between 1991 and 2006 [8] and 61 patients with 77 meningiomas and a median volume of 5.6 cc were treated between 1991 and 2013 [51]. The actuarial tumour control rate was 70% at 5 years [8]. In both cohorts, new or worsened peri-tumoural oedema occurred in 40% [8, 51] (40.4% [8]) with 8.2% being symptomatic in the earlier cohort [8]. Post-radiosurgery seizures were seen in 14.3% including 6% patients who had not experienced any seizures prior to radiosurgery [8]. The median interval between GKRS and oedema peak ranged between 6 and 24 months with a median at 18 months [51]. Tumour volume above 10 cc and prior existing peri-tumoural oedema were factors for new or worsening oedema [51]. Hasegawa reported a similar rate of symptomatic radiation-induced oedema in 7% of convexity, parasagittal or falcine meningiomas undergoing Gamma Knife radiosurgery with actuarial 5- and 10-year local tumour control rates of 87% and 71%, respectively [15]. Radiosurgery of parasagittal and parafalcine meningiomas may be associated with a higher risk for the development of radiation-induced oedema, and hence, the volume of radiosurgically treated tumours in this region should be consequently restricted. The necessity of prior resection has to be scrutinized with radiosurgical options remaining for smaller or unresectable tumours, for postoperative tumour remnants and meningiomas with sinus invasion.

### Planned surgical/radiosurgical cooperation

As meningiomas are frequently large at diagnosis and the risk profile of radiosurgery is significantly increased in large tumours, prior surgical resection and volume reduction generally create the prerequisites for a successful long-term outcome. Since, however, incomplete resections carry a significant risk for recurrence, a completing postoperative radiosurgical treatment is often necessary. From a surgical point of view, the necessity for a postoperative treatment is occasionally questioned since benign meningiomas develop at variable growth rates with often low doubling times [22, 24] and significant growth of postoperative tumour remnants may not always be noticed in a shorter follow-up. As a result, the surgical approach to tumour remnants that are deemed unresectable is often a ‘wait-and-see’ attitude with a potential additional surgical removal only if the re-growing tumour has reached a clinically relevant mass effect.

The risk for long-term growth, however, is significant: tumour growth occurred in 76% of petroclival meningiomas after a median follow-up of 85 months with associated functional deterioration in 63% of cases when tumours were growing [60]. Similarly, Mathiesen and colleagues had shown that after deliberate non-radical surgery (Simpson grade IV), tumour recurrences appeared in 72% [40], while a combined treatment of immediate Gamma Knife radiosurgery after a tailored microsurgical resection provided a low recurrence rate of 10% [40]. The documented long-term tumour control achieved by stereotactic radiosurgery has contributed to a policy change in open resective neurosurgery as tumour components close to functional brain or cranial nerves can generally be left in place during surgery and can later be treated with radiosurgery, thus reducing the need for an aggressive resection [1, 3, 42].

Since the tumour volume is a significantly complicating factor for stereotactic radiosurgery, it would not be beneficial to monitor a meningioma without treatment until the tumour progresses significantly. Hence, from a radiosurgical point of view, any radiosurgical treatment should be carried out while tumours are still as small as possible.

## Conclusions

The current retrospective analysis provides a 10-year follow-up and comprises one of the longest available follow-up studies of radiosurgically treated meningiomas. The current series documents a persistent high local tumour control and a persistently low risk for side effects after Gamma Knife treatment, while providing an estimation of a necessary minimum dose for long-term tumour control in meningiomas. The study confirms the validity of previous short-term data in a long-term perspective. In critical locations, stereotactic radiosurgery can replace a complicated surgical resection. In a planned surgical/radiosurgical cooperation, the need for an aggressive tumour resection is reduced as stereotactic radiosurgery provides a documented long-term control of tumour remnants.

## References

[1] Aboukais R, Zairi F, Reyns N, Le Rhun E, Touzet G, Blond S, Lejeune JP (2014). Surgery followed by radiosurgery: a deliberate valuable strategy in the treatment of intracranial meningioma. Clin Neurol Neurosurg.

[2] Aichholzer M, Bertalanffy A, Dietrich W, Roessler K, Pfisterer W, Ungersboeck K, Heimberger K, Kitz K (2000). Gamma knife radiosurgery of skull base meningiomas. Acta Neurochir.

[3] Black PM, Villavicencio AT, Rhouddou C, Loeffler JS (2001). Aggressive surgery and focal radiation in the management of meningiomas of the skull base: preservation of function with maintenance of local control. Acta Neurochir.

[4] Cohen-Inbar O, Lee CC, Schlesinger D, Xu Z, Sheehan JP (2016). Long-term results of stereotactic radiosurgery for skull base meningiomas. Neurosurgery.

[5] Cohen-Inbar O, Tata A, Moosa S, Lee CC, Sheehan JP (2018). Stereotactic radiosurgery in the treatment of parasellar meningiomas: long-term volumetric evaluation. J Neurosurg.

[6] Couldwell WT, Fukushima T, Giannotta SL, Weiss MH (1996). Petroclival meningiomas: surgical experience in 109 cases. J Neurosurg.

[7] Ding D, Starke RM, Kano H, Nakaji P, Barnett GH, Mathieu D, Chiang V, Omay SB, Hess J, McBride HL, Honea N, Lee JY, Rahmathulla G, Evanoff WA, Alonso-Basanta M, Lunsford LD, Sheehan JP (2014). Gamma knife radiosurgery for cerebellopontine angle meningiomas: a multicenter study. Neurosurgery.

[8] Ding D, Xu Z, McNeill IT, Yen CP, Sheehan JP (2013). Radiosurgery for parasagittal and parafalcine meningiomas. J Neurosurg.

[9] Flannery TJ, Kano H, Lunsford LD, Sirin S, Tormenti M, Niranjan A, Flickinger JC, Kondziolka D (2010). Long-term control of petroclival meningiomas through radiosurgery. J Neurosurg.

[10] Flickinger JC, Kondziolka D, Maitz AH, Lunsford LD (2003). Gamma knife radiosurgery of imaging-diagnosed intracranial meningioma. Int J Radiat Oncol Biol Phys.

[11] Gande A, Kano H, Bowden G, Mousavi SH, Niranjan A, Flickinger JC, Lunsford LD (2014). Gamma Knife radiosurgery of olfactory groove meningiomas provides a method to preserve subjective olfactory function. J Neurooncol.

[12] Ganz JC (2014). Stockholm radiosurgery developing 1968-1982. Prog Brain Res.

[13] Hafez RF, Morgan MS, Fahmy OM (2015). Stereotactic Gamma Knife surgery safety and efficacy in the management of symptomatic benign confined cavernous sinus meningioma. Acta Neurochir (Wien).

[14] Harrison G, Kano H, Lunsford LD, Flickinger JC, Kondziolka D (2016). Quantitative tumor volumetric responses after Gamma Knife radiosurgery for meningiomas. J Neurosurg.

[15] Hasegawa T, Kida Y, Yoshimoto M, Iizuka H, Ishii D, Yoshida K (2011). Gamma Knife surgery for convexity, parasagittal, and falcine meningiomas. J Neurosurg.

[16] Hasegawa T, Kida Y, Yoshimoto M, Koike J, Iizuka H, Ishii D (2007). Long-term outcomes of Gamma Knife surgery for cavernous sinus meningioma. J Neurosurg.

[17] Hayashi M, Chernov M, Tamura N, Izawa M, Muragaki Y, Iseki H, Okada Y, Takakura K (2011). Gamma knife robotic microradiosurgery for benign skull base meningiomas: tumor shrinkage may depend on the amount of radiation energy delivered per lesion volume (unit energy). Stereotact Funct Neurosurg.

[18] Hoe Y, Choi YJ, Kim JH, Kwon do H, Kim CJ, Cho YH (2015). Peritumoral brain edema after stereotactic radiosurgery for asymptomatic intracranial meningiomas: risks and pattern of evolution. J Korean Neurosurg Soc.

[19] Igaki H, Maruyama K, Koga T, Murakami N, Tago M, Terahara A, Shin M, Nakagawa K, Ohtomo K (2009). Stereotactic radiosurgery for skull base meningioma. Neurol Med Chir (Tokyo).

[20] Iwai Y, Yamanaka K, Ikeda H (2008). Gamma Knife radiosurgery for skull base meningioma: long-term results of low-dose treatment. J Neurosurg.

[21] Iwai Y, Yamanaka K, Ishiguro T (2003). Gamma knife radiosurgery for the treatment of cavernous sinus meningiomas. Neurosurgery.

[22] Jaaskelainen J, Haltia M, Laasonen E, Wahlstrom T, Valtonen S (1985). The growth rate of intracranial meningiomas and its relation to histology. An analysis of 43 patients. Surg Neurol.

[23] Jang CK, Jung HH, Chang JH, Chang JW, Park YG, Chang WS (2015). Long-term results of Gamma Knife radiosurgery for intracranial meningioma. Brain Tumor Res Treat.

[24] Jung HW, Yoo H, Paek SH, Choi KS (2000). Long-term outcome and growth rate of subtotally resected petroclival meningiomas: experience with 38 cases. Neurosurgery.

[25] Kaprealian T, Raleigh DR, Sneed PK, Nabavizadeh N, Nakamura JL, McDermott MW (2016). Parameters influencing local control of meningiomas treated with radiosurgery. J Neurooncol.

[26] Kim M, Cho YH, Kim JH, Kim CJ, Kwon DH (2017). Analysis the causes of radiosurgical failure in intracranial meningiomas treated with radiosurgery. Clin Neurol Neurosurg.

[27] Kobayashi T, Kida Y, Mori Y (2001). Long-term results of stereotactic gamma radiosurgery of meningiomas. Surg Neurol.

[28] Kollova A, Liscak R, Novotny J, Vladyka V, Simonova G, Janouskova L (2007). Gamma Knife surgery for benign meningioma. J Neurosurg.

[29] Kondziolka D, Flickinger JC, Perez B (1998). Judicious resection and/or radiosurgery for parasagittal meningiomas: outcomes from a multicenter review. Gamma Knife Meningioma Study Group. Neurosurgery.

[30] Kondziolka D, Patel AD, Kano H, Flickinger JC, Lunsford LD (2016). Long-term outcomes after Gamma Knife radiosurgery for meningiomas. Am J Clin Oncol.

[31] Kreil W, Luggin J, Fuchs I, Weigl V, Eustacchio S, Papaefthymiou G (2005). Long term experience of gamma knife radiosurgery for benign skull base meningiomas. J Neurol Neurosurg Psychiatry.

[32] Kuhn EN, Taksler GB, Dayton O, Loganathan A, Bourland D, Tatter SB, Laxton AW, Chan MD (2014). Is there a tumor volume threshold for postradiosurgical symptoms? A single-institution analysis. Neurosurgery.

[33] Kunz WG, Jungblut LM, Kazmierczak PM, Vettermann FJ, Bollenbacher A, Tonn JC, Schichor C, Rominger A, Albert NL, Bartenstein P, Reiser MF, Cyran CC (2017). Improved detection of transosseous meningiomas using (68)Ga-DOTATATE PET/CT compared with contrast-enhanced MRI. J Nucl Med.

[34] Leavitt JA, Stafford SL, Link MJ, Pollock BE (2013). Long-term evaluation of radiation-induced optic neuropathy after single-fraction stereotactic radiosurgery. Int J Radiat Oncol Biol Phys.

[35] Lee JY, Kondziolka D, Flickinger JC, Lunsford LD (2007). Radiosurgery for intracranial meningiomas. Prog Neurol Surg.

[36] Lee S, Kwon do H, Kim CJ, Kim JH (2016). Long-term outcomes following Gamma Knife radiosurgery for small, newly diagnosed meningiomas. Clin Neurol Neurosurg.

[37] Li D, Tang J, Ren C, Wu Z, Zhang LW, Zhang JT (2016). Surgical management of medium and large petroclival meningiomas: a single institution’s experience of 199 cases with long-term follow-up. Acta Neurochir (Wien).

[38] Maruyama K, Shin M, Kurita H, Kawahara N, Morita A, Kirino T (2004). Proposed treatment strategy for cavernous sinus meningiomas: a prospective study. Neurosurgery.

[39] Mathiesen T, Lindquist C, Kihlstrom L, Karlsson B (1996). Recurrence of cranial base meningiomas. Neurosurgery.

[40] Mathiesen T, Pettersson-Segerlind J, Kihlstrom L, Ulfarsson E (2014). Meningiomas engaging major venous sinuses. World Neurosurg.

[41] Morita A, Coffey RJ, Foote RL, Schiff D, Gorman D (1999). Risk of injury to cranial nerves after gamma knife radiosurgery for skull base meningiomas: experience in 88 patients. J Neurosurg.

[42] Nanda A, Thakur JD, Sonig A, Missios S (2016). Microsurgical resectability, outcomes, and tumor control in meningiomas occupying the cavernous sinus. J Neurosurg.

[43] Natarajan SK, Sekhar LN, Schessel D, Morita A (2007). Petroclival meningiomas: multimodality treatment and outcomes at long-term follow-up. Neurosurgery.

[44] Nicolato A, Foroni R, Alessandrini F, Maluta S, Bricolo A, Gerosa M (2002). The role of Gamma Knife radiosurgery in the management of cavernous sinus meningiomas. Int J Radiat Oncol Biol Phys.

[45] Park SH, Kano H, Niranjan A, Flickinger JC, Lunsford LD (2014). Stereotactic radiosurgery for cerebellopontine angle meningiomas. J Neurosurg.

[46] Patibandla MR, Lee CC, Sheehan J (2017). Stereotactic radiosurgery of central skull base meningiomas-volumetric evaluation and long-term outcomes. World Neurosurg.

[47] Pollock BE, Stafford SL, Link MJ, Garces YI, Foote RL (2012). Single-fraction radiosurgery for presumed intracranial meningiomas: efficacy and complications from a 22-year experience. Int J Radiat Oncol Biol Phys.

[48] Roche PH, Regis J, Dufour H, Fournier HD, Delsanti C, Pellet W, Grisoli F, Peragut JC (2000). Gamma knife radiosurgery in the management of cavernous sinus meningiomas. J Neurosurg.

[49] Santacroce A, Walier M, Regis J, Liscak R, Motti E, Lindquist C, Kemeny A, Kitz K, Lippitz B, Martinez Alvarez R, Pedersen PH, Yomo S, Lupidi F, Dominikus K, Blackburn P, Mindermann T, Bundschuh O, van Eck AT, Fimmers R, Horstmann GA (2012). Long-term tumor control of benign intracranial meningiomas after radiosurgery in a series of 4565 patients. Neurosurgery.

[50] Sheehan JP, Cohen-Inbar O, Ruangkanchanasetr R, Bulent Omay S, Hess J, Chiang V, Iorio-Morin C, Alonso-Basanta M, Mathieu D, Grills IS, Lee JY, Lee CC, Dade Lunsford L (2015). Post-radiosurgical edema associated with parasagittal and parafalcine meningiomas: a multicenter study. J Neurooncol.

[51] Sheehan JP, Lee CC, Xu Z, Przybylowski CJ, Melmer PD, Schlesinger D (2015). Edema following Gamma Knife radiosurgery for parasagittal and parafalcine meningiomas. J Neurosurg.

[52] Sheehan JP, Starke RM, Kano H, Barnett GH, Mathieu D, Chiang V, Yu JB, Hess J, McBride HL, Honea N, Nakaji P, Lee JY, Rahmathulla G, Evanoff WA, Alonso-Basanta M, Lunsford LD (2015). Gamma Knife radiosurgery for posterior fossa meningiomas: a multicenter study. J Neurosurg.

[53] Sheehan JP, Starke RM, Kano H, Barnett GH, Mathieu D, Chiang V, Yu JB, Hess J, McBride HL, Honea N, Nakaji P, Lee JY, Rahmathulla G, Evanoff WA, Alonso-Basanta M, Lunsford LD (2015). Gamma Knife radiosurgery for posterior fossa meningiomas: a multicenter study. J Neurosurg.

[54] Sheehan JP, Starke RM, Kano H, Kaufmann AM, Mathieu D, Zeiler FA, West M, Chao ST, Varma G, Chiang VL, Yu JB, McBride HL, Nakaji P, Youssef E, Honea N, Rush S, Kondziolka D, Lee JY, Bailey RL, Kunwar S, Petti P, Lunsford LD (2014). Gamma Knife radiosurgery for sellar and parasellar meningiomas: a multicenter study. J Neurosurg.

[55] Sindou M, Wydh E, Jouanneau E, Nebbal M, Lieutaud T (2007). Long-term follow-up of meningiomas of the cavernous sinus after surgical treatment alone. J Neurosurg.

[56] Skeie BS, Enger PO, Skeie GO, Thorsen F, Pedersen PH (2010). Gamma knife surgery of meningiomas involving the cavernous sinus: long-term follow-up of 100 patients. Neurosurgery.

[57] Starke R, Kano H, Ding D, Nakaji P, Barnett GH, Mathieu D, Chiang V, Yu JB, Hess J, McBride HL, Honea N, Lee JY, Rahmathulla G, Evanoff WA, Alonso-Basanta M, Lunsford LD, Sheehan JP (2014). Stereotactic radiosurgery of petroclival meningiomas: a multicenter study. J Neurooncol.

[58] Starke RM, Przybylowski CJ, Sugoto M, Fezeu F, Awad AJ, Ding D, Nguyen JH, Sheehan JP (2015). Gamma Knife radiosurgery of large skull base meningiomas. J Neurosurg.

[59] Starke RM, Williams BJ, Hiles C, Nguyen JH, Elsharkawy MY, Sheehan JP (2012). Gamma knife surgery for skull base meningiomas. J Neurosurg.

[60] Van Havenbergh T, Carvalho G, Tatagiba M, Plets C, Samii M (2003). Natural history of petroclival meningiomas. Neurosurgery.

[61] Zachenhofer I, Wolfsberger S, Aichholzer M, Bertalanffy A, Roessler K, Kitz K, Knosp E (2006). Gamma-knife radiosurgery for cranial base meningiomas: experience of tumor control, clinical course, and morbidity in a follow-up of more than 8 years. Neurosurgery.

